# The clonal evolution of two distinct T315I-positive BCR-ABL1 subclones in a Philadelphia-positive acute lymphoblastic leukemia failing multiple lines of therapy: a case report

**DOI:** 10.1186/s12885-017-3511-2

**Published:** 2017-08-05

**Authors:** Caterina De Benedittis, Cristina Papayannidis, Claudia Venturi, Maria Chiara Abbenante, Stefania Paolini, Sarah Parisi, Chiara Sartor, Michele Cavo, Giovanni Martinelli, Simona Soverini

**Affiliations:** Department of Experimental, Diagnostic and Specialty Medicine, Institute of Hematology “L. e A. Seràgnoli”, University of Bologna, Via Massarenti, 9-40138 Bologna, Italy

**Keywords:** BCR-ABL1 mutation, T315I mutation, Ph+ Acute Lymphoblastic Leukemia, Resistance, Case Report, Relapse, Dasatinib, Ponatinib, Transplant, Blinatumomab

## Abstract

**Background:**

The treatment of Philadelphia chromosome-positive Acute Lymphoblastic Leukemia (Ph+ ALL) patients who harbor the T315I BCR-ABL1 mutation or who have two or more mutations in the same BCR-ABL1 molecule is particularly challenging since first and second-generation Tyrosine Kinase Inhibitors (TKIs) are ineffective. Ponatinib, blinatumomab, chemotherapy and transplant are the currently available options in these cases.

**Case presentation:**

We here report the case of a young Ph+ ALL patient who relapsed on front-line dasatinib therapy because of two independent T315I-positive subclones, resulting from different nucleotide substitutions -one of whom never reported previously- and where additional mutant clones outgrew and persisted despite ponatinib, transplant, blinatumomab and conventional chemotherapy. Deep Sequencing (DS) was used to dissect the complexity of BCR-ABL1 kinase domain (KD) mutation status and follow the kinetics of different mutant clones across the sequential therapeutic approaches.

**Conclusions:**

This case presents several peculiar and remarkable aspects: i) distinct clones may acquire the same amino acid substitution via different nucleotide changes; ii) the T315I mutation may arise also from an ‘act’ to ‘atc’ codon change; iii) the strategy of temporarily replacing TKI therapy with chemo or immunotherapy, in order to remove the selective pressure and deselect aggressive mutant clones, cannot always be expected to be effective; iv) BCR-ABL1-mutated sub-clones may persist at very low levels (undetectable even by Deep Sequencing) for long time and then outcompete BCR-ABL1-unmutated ones becoming dominant even in the absence of any TKI selective pressure.

**Electronic supplementary material:**

The online version of this article (doi:10.1186/s12885-017-3511-2) contains supplementary material, which is available to authorized users.

## Background

First and second-generation Tyrosine Kinase Inhibitors approved for the treatment of Philadelphia-chromosome positive Acute Lymphoblastic Leukemia have Achilles heels that the BCR-ABL1 target oncoprotein may exploit to escape from inhibition: point mutations in the kinase domain may be selected that impair TKI binding [1–4]. Particularly challenging is the treatment of patients with the T315I mutation and of patients with multi-TKI resistant disease who harbor two or more mutations in the same BCR-ABL1 molecule (compound mutants), where first and second-generation TKIs are ineffective [5, 6]. Patients positive for the T315I mutation may now benefit from the third-generation TKI ponatinib, that has the ability to bind and inhibit every single kinase domain mutant [7]. It remains unclear whether ponatinib efficacy may be reduced by some compound mutants including the T315I plus another dasatinib or nilotinib-resistant mutation, since contrasting in vitro and in vivo data have been reported [8]. An alternative rescue strategy may be to ease the TKI selective pressure by switching to chemotherapy or immunotherapy whose efficacy should not be influenced by patient mutation status [9, 10]. We here report the case of a young patient who relapsed on front-line dasatinib therapy because of two independent T315I-positive subclones, resulting from different nucleotide substitutions never reported previously and where additional mutant clones outgrew and persisted despite ponatinib, transplant, blinatumomab and conventional chemotherapy. Deep Sequencing was used to dissect the complexity of BCR-ABL1 KD mutation status and follow the kinetics of different mutant clones across the sequential therapeutic approaches.

## Case presentation

**Fig. 1:**
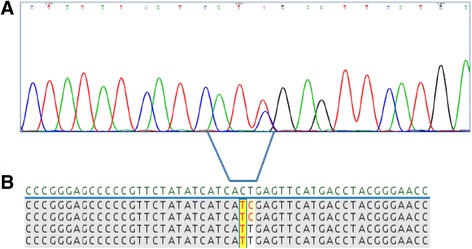
Example of clonal analysis for sample ALL-8. **a**) Conventional Sanger Sequencing results showing the double nucleotide substitution at codon 315; **b**) screenshot showing a portion of the global alignment of sequence reads obtained with AVA software, where codon 315 maps. Deep Sequencing allowed to resolve two distinct populations of mutants at this codon, one harboring the T315I (att) and one harboring the T315I (atc). Sequence were compared to the wild-type sequence (*green* at the top) using BLAST, GenBank Accession Number X16416

**Fig. 2:**
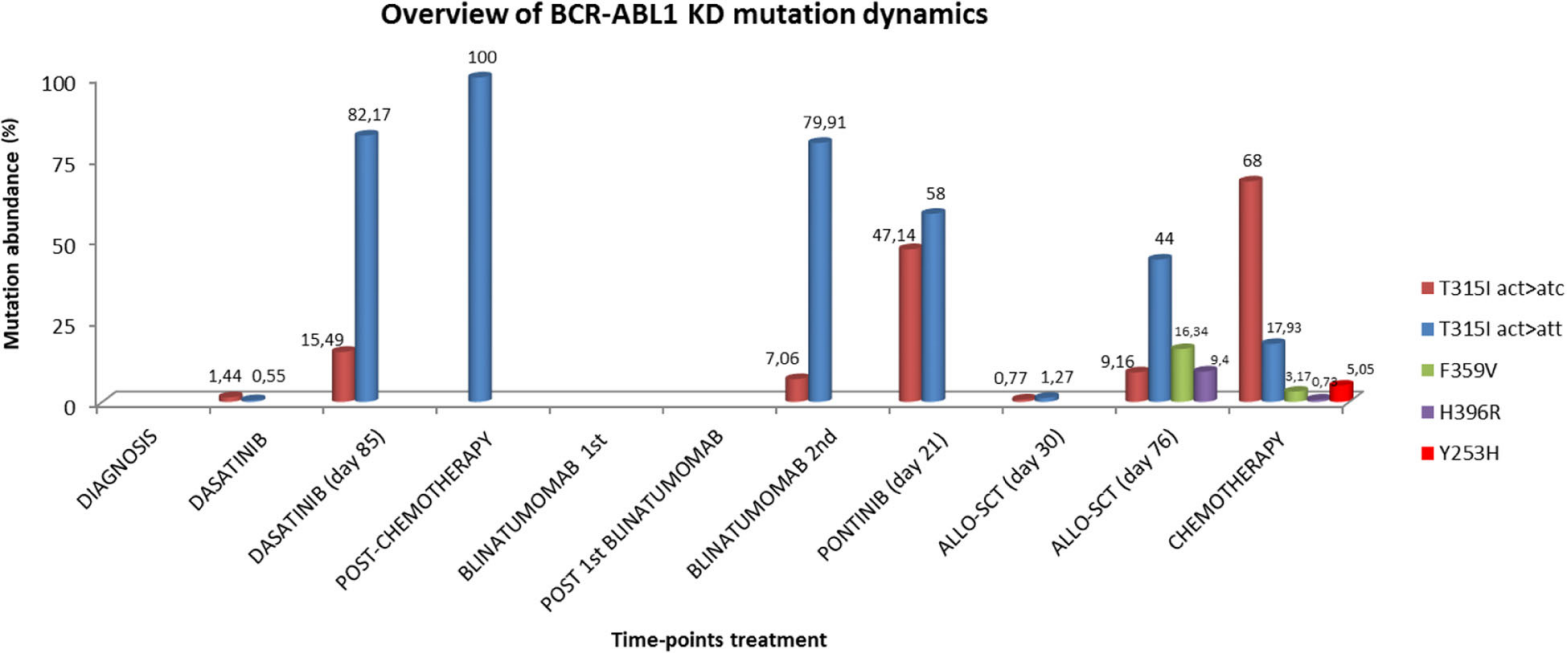
Overview of BCR-ABL1 KD mutations dynamics and their relative frequency at different time-points during treatment. Graphical illustration of the kinetics of mutated population abundances for each time points in relation to therapeutic intervention

**Fig. 3:**
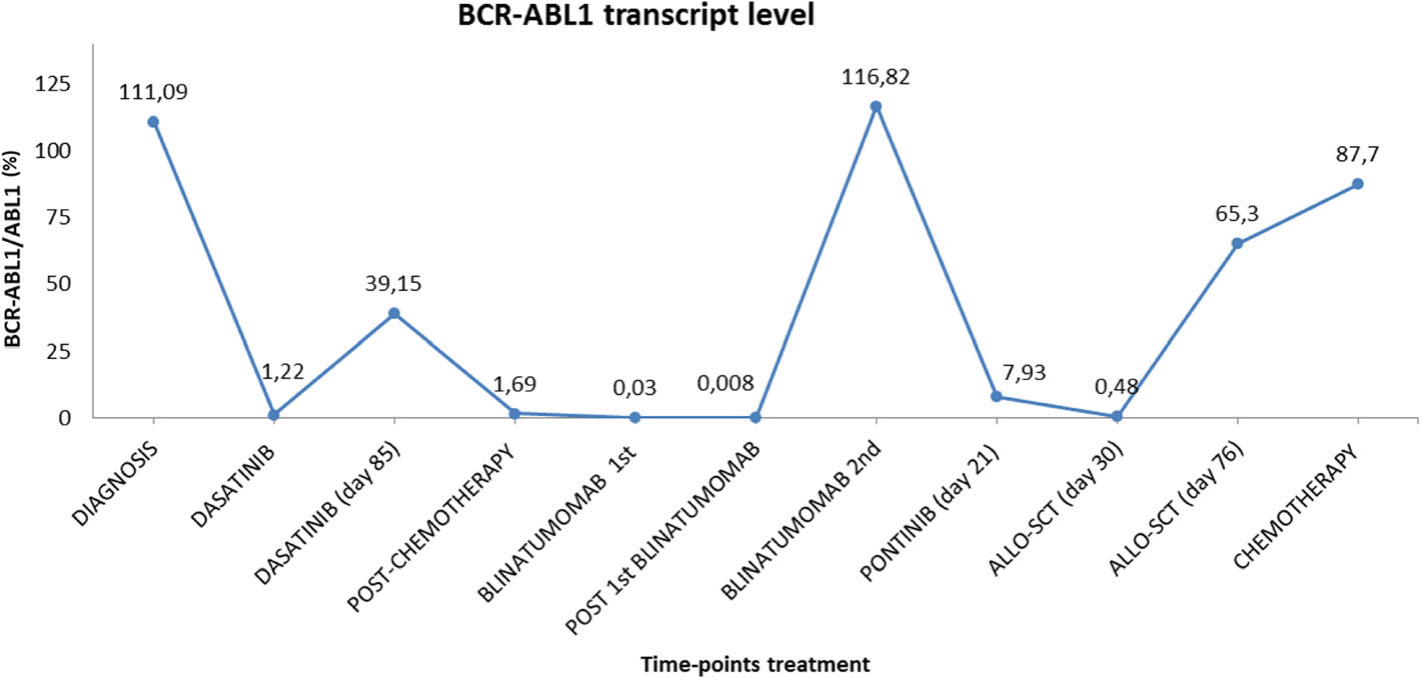
Overview of BCR-ABL1 transcript levels at different time-points during treatment. Graphical illustration of the BCR-ABL1 transcript levels for each time-points in relation to therapeutic intervention assessed by real-time quantitative RT-PCR

The patient was a 19-years-old man who, despite very good clinical conditions, presented with abnormal peripheral blood counts before receiving a scheduled surgical procedure. Physical examination revealed only a moderate splenomegaly. He was hyperleucocytotic, with a peripheral WBC count of 40.7×10^9^/L, a normal hemoglobin value and a moderate thrombocytopenia. A bone marrow aspirate showed 88% lymphoblasts, expressing the CD19, CD10, CD22, CD34, CD58, and CD45 antigens. Chromosome banding analysis of bone marrow revealed a normal karyotype; however, polymerase chain reaction revealed the BCR-ABL1 e1a2 fusion transcript. BCR-ABL1 transcript level assessed by real-time quantitative RT-PCR (RQ-PCR) was 111.09%. No involvement of central nervous system was detected, since all lumbar punctures performed showed the absence of leukemic cells in cerebrospinal fluid. Thus, a diagnosis of BCR-ABL1 (p190)-positive B-ALL was made. After informed consent was obtained, treatment was initiated according to the GIMEMA LAL1509 clinical trial that included a steroid-based pre-phase, followed by dasatinib induction therapy at 140 mg/daily for 84 days. After 52 days of dasatinib therapy, the patient obtained a complete hematological response with a mild decrease of the BCR-ABL1 transcript levels (1.22%). At day 85 the patient unfortunately progressed. Conventional Sanger Sequencing analysis showed evidence of a C to T nucleotide substitution at position 1091 of the ABL1 sequence in a proportion of BCR-ABL1 transcripts, resulting in the dasatinib-resistant T315I mutation. According to protocol schedule, the patient was treated with one course of standard chemotherapy, consisting of clofarabine 80 mg/daily, for 5 days and cyclophosphamide 800 mg/daily, for 5 days. The patient achieved complete hematological remission, but persistence of the BCR-ABL1 fusion transcript at the molecular level was observed (1.69%). A severe cardiac toxicity contraindicated the administration of an additional course of consolidation chemotherapy. Therefore, because of the minimal residual disease persistence, the patient was enrolled in the MT103–203 clinical study of blinatumomab, an anti-CD19 T cell engager antibody, as continuous intravenous infusion for 28-days cycles. After one course of treatment with 15 mcg/sqm/daily, which was well tolerated, a brilliant response was observed: the BCR-ABL1 transcript level significantly decreased down to 0.008% and the T315I mutation disappeared at conventional sequencing analysis. In consideration of the persistently suboptimal heart function, which would have seriously compromised the outcome of a transplantation procedure, a second course of therapy with blinatumomab was then started two weeks after the end of the first one, as required by protocol schedule. Unfortunately, the patient suddenly and unexpectedly relapsed after 10 days, with a remarkable hyperleucocytosis and a high percentage of lymphoblasts, with the same immunophenotypic signature detected at diagnosis. Conventional Sanger Sequencing showed that the T315I mutation had reappeared in a proportion of BCR-ABL1 transcripts. Salvage therapy with ponatinib at the dosage of 45 mg/daily was immediately started, but despite a very good tolerance to the compound, only a hematological improvement was observed, without significant changes in BCR-ABL1 transcript levels. At this timepoint, conventional Sanger Sequencing analysis displayed an unusual pattern of nucleotide substitutions: a C to T substitution at position 1091 and a T to C substitution at the adjacent 1092 position, suggesting the presence of two clones that could not be further characterized. Deep Sequencing was then performed as detailed in Soverini et al. [11]; a median of 4166 (range, 2519–10,297) high quality reads were obtained across the different runs. The analysis demonstrated that two distinct T315I-positive subclones were coexisisting: one subclone, with a relative abundance of 58%, had the usual ‘act’ to ‘att’ codon shift resulting from the c.1091c>t nucleotide change, whereas the other one, with a relative abundance of 47.14%, had both a c.1091c>t and a c.1092 t>c nucleotide change, thus leading to an ‘act’ to ‘atc’ codon shift still translating into a threonine to isoleucine amino acid change at position 315 (Fig. 1). Since TKI resistant BCR-ABL1 mutations existing prior to exposure may exist, we looked for the T315I mutation at diagnosis prior to dasatinib start, but Deep Sequencing didn’t find evidence. Deep Sequencing was then used to retrospectively investigate all the previous samples and revealed that the two distinct T315I-positive subclones were detectable since day 52 of dasatinib therapy, where both were identified at very low level: T315I act>att 0.56% and T315I act>atc 1.44%. At day 85, when the patient had relapsed on dasatinib, the proportions of the two clones had increased to 82,17% and 15.49% respectively. After chemotherapy, the T315I act>att subclone accounted for 100% of BCR-ABL1 transcripts, and then became undetectable, too, after the first course of blinatumomab. However, both subclones had quickly become detectable again by Deep Sequencing (act>att 79.91%; act>atc 7.06%) when the patient had relapsed during the second cycle. In the presence of a matched-related stem cell donor, and in the absence of further available therapeutic tools, the patient underwent allogeneic transplantation, with a conditioning regimen consisting of fludarabine, busulfan and thiotepa, in addition to ATG as graft-versus-host disease prophylaxis. A total of 16.12×10^8^/kg nucleated cells were infused, including 7.4×10^6^/kg CD34+ cells. No signs or symptoms of graft-versus-host disease occurred, and a full recovery on peripheral blood was observed after 16 days from transplantation. After one month from this procedure, the bone marrow evaluation showed a complete morphological remission. FISH analysis revealed an almost complete full donor chimerism; BCR-ABL1 transcript level was 0.48%. Although the mutation analysis performed by conventional sequencing did not show evidence of mutations, the greater sensitivity of Deep Sequencing allowed to identify, again, both the T315I-positive subclones at very low levels (act>att 1.27%; act>atc 0.77%). After 3 months from allogeneic stem cell transplantation, despite good clinical conditions and in the absence of symptoms of leukemia progression, the patient developed hyperleukocytosis, with mild anemia and thrombocytopenia. Bone marrow analysis showed a full hematological relapse, confirmed by a marked increase in BCR-ABL1 fusion transcripts (65.3%). The Deep Sequencing analysis performed at this time point showed the quick regrowth of both T315I-mutated subclones (act>att 44%; act>atc 9.16%). Unexpectedly, the first clone was found to harbor additional point mutations: the F359V (16.34%) and H396R (9.47%). The rapid worsening of peripheral hematological values required an immediate therapeutic intervention. Therefore, after a brief and ineffective course of steroids and 6-mercaptopurine and hydroxyurea as cytoreductive agents, the patient received salvage chemotherapy according to HAM schedule with high dose of cytarabine and mitoxantrone. Treatment was well tolerated, but subsequent iatrogenic bone marrow aplasia was complicated by a severe pulmonary infection, with microbiological and radiological images diagnostic for an invasive fungal infection. Therefore, the patient received dual antimycotic therapy with voriconazole and liposomal amphotericin b, achieving a significant improvement of imaging reports after 3 weeks of treatment. Bone marrow examination, which was performed one month after the end of chemotherapy, showed the persistence of a relevant amount of CD19+ lymphoid blast cells and BCR-ABL1 transcript level had further increased (87.8%). Deep Sequencing analysis showed again all the sub-clones previously identified T315I act>att (17.93%), T315I act>atc (67%), F359V (3.17%), H396R (0.73%). At this time point we observed the emergence of the Y253H point mutation, with an abundance of 5.05%. Two T315I-inclusive compound mutations were observed: T315I act>att + F359V (0.67%) and the T315I act>atc + Y253H (3.98%) (Fig. 2). In the following days, due to the onset of specific symptoms, related with leukemic progression, mainly represented by fever and lumbar pain, the patient received further cytoreductive chemotherapy, obtaining a partial response. Unfortunately, the patient developed a severe fungal pulmonary infection, and he died two months after, in progression disease. An overview of the BCR-ABL1 transcript levels, for each time point, assessed by real-time quantitative RT-PCR is reported in Fig. 3. Results of BCR-ABL1 KD mutation analysis performed by conventional Sanger sequencing and Deep Sequencing are reported in Additional file 1 Table S1.

## Discussion and conclusions

The case herein reported presents several peculiar and remarkable aspects. First of all, this patient developed two distinct dasatinib-resistant subclones, where the same T315I amino acid substitution was acquired via different nucleotide changes – a phenomenon of ‘convergent evolution’ that once again underlines how Darwinian theories well apply to cancer [12]. Notably, in one of the two subclones the T315I resulted from a previously unreported ‘act’ to ‘atc’ codon change, which requires two nucleotide substitutions. Whether the ‘atc’ subclone arose from a ‘ct’ to ‘tc’ dinucleotide change, or rather derived from the ‘T315I canonical’ ‘att’ mutant clone after a ‘t’ to ‘c’ mutation at the third codon position, is impossible to tell. However, the fact that the subclones were first detected by Deep Sequencing after only 52 days of dasatinib treatment and, at that time, they had identical abundance, would suggest a simultaneous independent origin. Both the T315I-positive subclones quickly became undetectable, even by Deep Sequencing, after only one course of blinatumomab but they even more quickly re-emerged during the second course – although blinatumomab is likely to be equally active against B-cells harboring mutated or unmutated BCR-ABL1. Interestingly, the T315I-positive clones persisted during ponatinib therapy, which was ineffective. Most likely, these two clones happened to carry some cellular or molecular mechanism of resistance to ponatinib, which became the real driver. Allogeneic hematopoietic stem cell transplantation failed to deplete the BCR-ABL1 mutated clones. After transplantation, in the absence of any kind of therapy, the patient quickly relapsed with the re-emergence of the two T315I-positive subclones. Even more inexplicably, additional BCR-ABL1 kinase domain mutations became detectable in the same or different subclones during subsequent salvage chemotherapy. The emergence of several T315I-inclusive compound mutations was observed after 3 months from allogeneic transplantation. When did they arise? Recent in vitro studies have shown that accumulation of more than one mutation within the same allele may be associated with increased oncogenic potential. They have also suggested that some T315I-inclusive compound mutants are highly resistant to all second-generation TKIs and not always fully sensitive to ponatinib [8]. It can be hypothesized that the mutants newly detected after transplant and after subsequent salvage chemotherapy indeed originated in very few Ph+ cells during ponatinib therapy, though they did not have the time to outgrow and become detectable by Deep Sequencing. It may even be hypothesized that they originated earlier, during dasatinib therapy, or present since diagnosis in very few Ph+ cells. In conclusion, we observed that the T315I mutation may be acquired via different nucleotide changes - also from an’ act’ to ‘atc’ codon change- and may persist despite ponatinib or transplant. In addition the strategy of temporarily replacing TKI therapy with chemo or immunotherapy, in order to remove the selective pressure and deselect aggressive mutant clones, cannot always be expected to be effective. The BCR-ABL1-mutated sub-clones may persist at very low levels for long time and then outcompete BCR-ABL1-unmutated ones becoming dominant even in the absence of any TKI selective pressure.

## Additional files

Additional file 1: Comparison between mutations detected by conventional Sanger sequencing and Deep sequencing and estimated clonal composition of the samples. Mutation-relative abundance of conventional Sanger Sequencing results was assessed on the basis of variant peak height. In the TKI/treatment column, the TKI or the treatment being administered at the time of analysis is indicated. In sample ALL-8 and 11, “T315?” denotes that 2 overlapping peaks at adjacent positions (c/t at 1091 and t/c at 1092) of codon 315 were identified in the Sanger Sequencing chromatogram and the resulting amino acid substitution(s) could not be resolved. (PDF 209 kb)

## Abbreviations

BCR-ABL1 KD: BCR-ABL1 kinase domain
DS: Deep Sequencing
Ph+ ALL: Philadelphia-chromosome positive acute lymphoblastic leukemia
RQ-PCR: Real-time quantitative RT-PCR
SS: Sanger sequencing
TKIs: Tyrosine kinase inhibitors
