# Supplementary material for: The clonal evolution of two distinct T315I-positive BCR-ABL1 subclones in a Philadelphia-positive acute lymphoblastic leukemia failing multiple lines of therapy: a case report
Source: BMC Cancer. 2017 Aug 5;17:523. doi: 10.1186/s12885-017-3511-2 (PMC5545087; doi:10.1186/s12885-017-3511-2)
Supplement: Supplementary file 1 — Comparison between mutations detected by conventional Sanger sequencing and Deep sequencing and estimated clonal composition of the samples. Mutation-relative abundance of conventional Sanger Sequencing results was assessed on the basis of variant peak height. In the TKI/treatment column, the TKI or the treatment being administered at the time of analysis is indicated. In sample ALL-8 and 11, “T315?” denotes that 2 overlapping peaks at adjacent positions (c/t at 1091 and t/c at 1092) of codon 315 were identified in the Sanger Sequencing chromatogram and the resulting amino acid substitution(s) could not be resolved. (PDF 209 kb) [file 12885_2017_3511_MOESM1_ESM.pdf]

Sample	Date	TKI/Treatment	BCR-ABL1/ABL1%	Mutations by SS	Mutations by DS	Compound
ALL-1	26/06/2013	DIAGNOSIS	111,09	WT	WT	
ALL-2	02/09/2013	DASATINIB<br>at140 mg/daily<br>(day 52)	1.22	WT	T315I (act>att) 0.56%<br>T315I (act>atc) 1.44%	
ALL-3	25/09/2013	DASATINIB<br>at140 mg/daily<br>(day 85)	39.15	T315I (act>att) ~60%	T315I (act>att) 82.17%<br>T315I (act>atc) 15.49%	
ALL-4	18/10/2013	POST – CHEMO<br>Clofarabine<br>(80 mg/daily. for 5 days)<br>Cyclophosphamide<br>(800 mg/daily. for 5 days)	1.69	T315I (act>att) 100%	T315I (act>att) 100%	
ALL-5	30/10/2013	BLINATUMOMAB<br>(CD19 antibody)	0.03	WT	WT	
ALL-6	11/12/2013	POST 1st COURSE<br>BLINATUMOMAB	0.008	WT	WT	
ALL-7	14/01/2014	2nd COURSE<br>BLINATUMOMAB	116.82	T315I (act>att) ~60%	T315I (act>att) 79.91%<br>T315I (act>atc) 7.06%	
ALL-8	05/02/2014	PONATINIB<br>45 mg/daily<br>(day 21)	7.93	T315?	T315I (act>att) 58%<br>T315I (act>atc) 47.14%	
ALL-9	16/04/2014	TRANSPLANT<br>(day 30)	0.48	WT	T315I (act>att) 1.27%<br>T315I (act>atc) 0.77%	
ALL-10	03/06/2014	TRANSPLANT<br>(day 76)	65.3	T315I (act>att) ~80%<br>F359V (ttc>gtc) ~20%	T315I (act>att) 44%<br>T315I (act>atc) 9.16%<br>F359V (ttc>gtc) 16.34%<br>H396R (cat>cgt) 9.47%	T315I (act>att) + F359V 16.34%<br>T315I (act>att) + H396R 9.47%
ALL-11	29/07/2014	CYTOREDUCTIVE<br>CHEMOTHERAPY	87.7	T315?<br>Y253H (tac>cac) ~20%	T315I (act>att) 17.93%<br>T315I (act>atc) 67%<br>Y253H (tac>cac) 5.05%<br>F359V (ttc>gtc) 3.17%<br>H396R (cat>cgt) 0.73%	T315I (act>atc) + Y253H 3.98%<br>T315I (act>att)+ F359V 0.67%
